# Association of Adherence to Diabetics Feeding Recommendation with Glycaemic Control and with Malnutrition Risk Among Normal Weight Persons with Type 2 Diabetes in Ghana

**DOI:** 10.21315/mjms2021.28.2.8

**Published:** 2021-04-21

**Authors:** Be-Ikuu Dominic Doglikuu, Abubakari Abdulai, Mehdi Yaseri, Elham Shakibazadeh, Abolghassem Djazayery, Khadijeh Mirzaei

**Affiliations:** 1 International Campus, Department of Community Nutrition, School of Nutritional Sciences and Dietetics, Tehran University of Medical Sciences, Tehran, Iran; 2 Department of Community Nutrition, School of Nutritional Sciences and Dietetics, Tehran University of Medical Sciences, Tehran, Iran; 3 Ministry of Health, Nursing and Midwifery Training College, TwifoPraso, Central Region, Ghana; 4 Department of Nutritional Sciences, School of Allied Health Sciences, University of Development Studies, Tamale, Ghana; 5 Department of Epidemiology and Biostatistics, School of Public Health, Tehran University of Medical Sciences, Tehran, Iran; 6 Department of Health Education and Promotion, School of Public Health, Tehran University of Medical Sciences, Tehran, Iran

**Keywords:** adherence, diabetes, feeding recommendation, glycaemic control, malnutrition-risk, Ghana

## Abstract

**Background:**

Diabetes mellitus (DM) is public health problem. Feeding-recommendations help persons with diabetes control glycaemia. The aim was to access the association between adherence to diabetics’ feeding recommendation with glycaemic control and with malnutrition risk.

**Methods:**

Cross-sectional study was conducted among 530 baseline normal weight (body mass index [BMI] 18.5 kg/m^2^–24.9 kg/m^2^) persons with type 2 diabetes (T2DM) in Brong Ahafo region of Ghana, from August 2018 to September 2019. Adherence to feeding recommendation was evaluated with perceived dietary adherence questionnaire (PDAQ). Malnutrition-risk was assessed using malnutrition universal screening tool. Multinomial logistics regression models were used to assess the association between adherence to diabetics’ feeding recommendation with glycaemic control and with malnutrition risk.

**Results:**

Participants were generally healthy. Weight (*P* = 0.011), total cholesterol (*P* = 0.003) and glycated haemoglobin (HbA1c)% (*P* < 0.001) were significant with adherence to diabetics feeding recommendation. Low adherence to diabetics’ feeding recommendation (adjusted odds ratio [AOR] 2.56; 95% CI: 1.44, 4.56; *P* < 0.001), low adherence to fruit and vegetables (AOR 2.71; 95% CI: 1.48, 4.99; *P* < 0.001), low adherence to whole grain, beans, starchy-fruits and plantain (AOR 3.29; 95% CI: 1.81, 6.02; *P* < 0.001), and low adherence to foods prepared with walnut, canola, sunflower, cotton seed and fish oils (AOR 2.62; 95% CI: 1.49, 4.58; *P* < 0.001) were significant with poor glycaemic control. Furthermore, low adherence to food prepared with walnut, canola, sunflower, cotton seed, fish or soy oils (AOR 0.54; 95% CI: 0.31, 0.95; *P* = 0.034) and low adherence to fish and lean meat (AOR 2.09; 95% CI: 1.14, 3.86; *P* = 0.017) were significant with moderate malnutrition risk.

**Conclusion:**

This study demonstrates that poor adherence feeding recommendation could be related to glycaemic control and malnutrition risk.

## Introduction

Diabetes mellitus (DM) is a global public health problem. It is a group of metabolic disorders characterised by elevated blood glucose over prolonged period of time. It is a serious medical condition that is very difficult to treat and expensive to manage thus contributing significant public health thread to people throughout the world. Globally it is estimated that 108 million adults had diabetes (i.e. 4.7% of the global population) in 1980 and was increased to 422 million (i.e. 8.5% of the global population) in 2014. By 2040, it is expected that DM will affect about 642 million adults worldwide (1).

Although DM was once seen as a disease of the affluence countries, due to rapid urbanisation, nutrition transition and sedentary lifestyles, its epidemiology has now changed to include many developing countries (2, 3). In sub-Saharan Africa, DM affects about 8 million people in the year 2000 and is projected to increase to 19 million by 2030 (4). In Ghana, diabetes prevalence study conducted between 1999 and 2011 showed increased prevalence of DM (5).

Although the mechanisms involve in driving DM prevalence in developing countries, are linked to nutrition transition, westernisation, urbanisation, technological advancement, food processing and food market globalisation (6), converging lines of evidences show that rapid transition from traditional lifestyles (energy intensive occupations, consumption of unrefined and low fat diets) to highly obesogenic lifestyles (more sedentary occupations, reduced physical activity, consumption of foods high in total calories, saturated fats, sugar and low in fibre) are largely responsible (7).

The health and economic ramification of DM to humans’ population is enormous. Ineffectively managed DM leads to increase risk of heart attacks, neuropathy, retinopathy and nephropathy which consequently cause poor blood circulation leading limbs amputation, blindness and kidney problems (8). Although early diagnosis and treatment through strict blood glucose, cholesterol management and pressure control, prevent the onset and progress of diabetic complications, adopting and maintaining healthy lifestyle behaviours such regular exercise, eating healthfully and maintaining healthy weight gain are important factors in decreasing risk associated with diabetics complication (9). Pharmaceutical formulations such as drugs are well known to significantly improve blood sugar, cholesterol and blood pressure (BP) among persons with DM, however, lifestyle modifications and feeding recommendation are fundamental factors in long term management of DM (10, 11).

Diabetics feeding recommendations are tailor made information design to guide persons with diabetes adopt to eating the healthiest foods in moderation, while sticking to regular meal times relative to medication intakes to control glycaemia (12, 13). Patients counselled to follow diabetics feeding recommendations are often asked to frequently choose and eat foods that contain whole grains, fruits and vegetables, and lean meat. Although dieticians periodically recommend these feeding recommendations to persons living with DM to follow in order to control glycaemia, little is known about how adherence to these feeding recommendations are associated with other adverse outcomes such as risk of malnutrition. People living with DM are vulnerable to other co-morbidities such as eating disorders, altered bowel conditions, dementia and depression. If one or more of these co-morbidities is/are present, patients’ adherence to diabetics feeding recommendations could be compromised and this could lead to other adverse outcomes including malnutrition risk. Despite this, little evidence is known about how adherence to of diabetics feeding recommendations is associated with malnutrition risk among people with DM. To demonstrate this, our study aims to investigate the association of adherence to diabetics’ feeding recommendation with glycaemic control and with malnutrition risk among normal weight persons living with type 2 diabetes mellitus (T2DM) in Brong Ahafo region, Ghana.

## Methods

### Study Design

Cross-sectional study was conducted among 530 baseline normal weight (body mass index [BMI] 18.5 kg/m^2^–24.9 kg/m^2^) persons living with T2DM in Brong Ahafo region, Ghana.

### Sample Size Determination

Single population proportion formula $\left(n=\frac{Z^{2}P(1-P)}{e^{2}}\right)$ was used to determine this study sample size. The letter *n* in the formula denotes the study sample size, *Z* denotes normal standard distribution of 1.96 for 95% confidence interval, *P* is the true population proportion of adherence to diabetics feeding recommendation in the study area (Brong Ahafo region) and *e* is standard error (5%). Previous study in Brong Ahafo region reported 68.5% adherence to diabetics feeding recommendation. Substituting these values in the equation above, the sample size *n* was calculated as $n=\frac{1.96^{2}\times 0.685(1-0.685)}{0.05^{2}}=332$. However, with the event of non-response and registration error, a contingency sample of 60% was considered in the sample, therefore the final sample was increased to 0.6 × 332 = 531.2 ≈ 532.

### Inclusion and Exclusion Criteria

Individuals aged 18 years old and above who were diagnosed with T2DM by physicians using the American Diabetes Association (ADA) Diagnostic and Classification Guideline 2011 (14), and counselled to follow diabetics feeding recommendations for at least 3 months and over were included in this study. Patients aged 70 years old and above who cannot answer interview questions; intellectually deficient, severely ill, and pregnant and lactating mothers were excluded from this study.

### Sampling Technique

Simple random sampling was used to select six hospitals in Brong Ahafo region and the eligible participants recruited using systematic sampling from patients’ registers.

## Dietary Intake Assessment

Participants were asked to respond to three separate 24 h dietary recall questionnaire (one on Monday, Wednesday and Saturday). This was done to evaluate participants’ dietary intakes in a typical day. Participants were asked to report details of all foods and drinks or beverages they took in each previous day prior to the interview. They were asked to report detail of the foods they ate, the preparation method, type of oil added to the foods, the portion size served and the actual quantity eaten. The information obtained was summed up and analysed using Ntri.IV software to obtain participants’ mean nutrients intakes.

## Anthropometry and Other Measurements

Participants’ age, diabetes duration, medications intakes and other demographic characteristics were collected using questionnaires. Weight (kg) and height (m) were measured and recorded to the nearest 0.5 kg and 0.5 m using adult weighing scale and stadiometer, respectively. In all the measurements, participants were asked to wear light clothes without shoes and were in standing position for measurement to be taken. BMI was calculated by dividing weight in kilograms by height in metres square. Systolic and diastolic BP were measured using manual sphygmomanometer and stethoscope, and the reading recorded to the nearest 0.5 mmHg after participants were allowed to relax for 5 min or more.

Participants’ physical activity (PA) level was measured using International Physical Activity short form Questionnaire (IPAQ) (15), and categorised into ‘low’ (< 600 metabolic equivalent (MET)/h per week), ‘moderate’ (between 600 MET/h and 3000 MET/h per week) and ‘high’ physical activity level (> 3000 MET/h per week) according to the IPAQ scores.

## Assessment of Adherence to Feeding Recommendation

Perceived Dietary Adherence Questionnaire (PDAQ) for people living with T2DM was used to assess adherence to diabetics feeding recommendation (16). This questionnaire is 9 items and 7-point Likert’s scale designed to elicit information about adherence to diabetic feeding recommendation from patients with DM. This 7-point likert’s scale questionnaire have points ranging from 0 to 7, where 0 means non-adherence at all and 7 means highest adherence to feeding recommendation. The 9 items in the questionnaire were summed up to form a global score in data analysis. Patients’ total adherence on the global score was 63. This score was ranged as low, moderate and high adherence based on patients’ score on the scale. Patients who scored 0 to 21 points on the scale were considered to have low adherence, those who scored from 22 to 42 were said to have moderate adherence and those who scored 43–63 points were said to have high adherence to feeding recommendation. The questionnaire was pretested among 20 participants (Cronbach’s alpha of 0.95) before using in the main study.

## Laboratory/Biochemistry Analysis

Whole blood sample was used to estimates glycated haemoglobin (HbA1c) by turbidimetric inhibition immunoassay method using Cobas Integra automated Chemistry analyser (Roche Cobas Integra 400 Plus, Roche Diagnostics, USA) (17). Participants’ overnight fasting blood sugar (FBS) samples were collected in ethylenediaminetetraacetic acid (EDTA) test tubes to prevent cross reaction prior to analysis. During the analysis, the well-mixed EDTA-anti-coagulated whole blood was transferred into sample test-tubes and placed on a rack. The red blood cells in the test tubes were latter haemolysed using low osmotic pressure and the free haemoglobin degraded by pepsin to liberate the N-terminal of the beta chain (β-N-terminal) of the HbA1c. The HbA1c β-N-terminal was then bound with latex particles-bound monoclonal antibodies while the remaining free antibodies were agglutinated using synthetic polymers. This process formed multiple copies of β-N-terminal structure of HbA1c and then leaves the test sample turbid. The change in turbidity of the sample was then measured at 552 nm and the final HbA1c value expressed in percentage using the formula: HbA1c (%) = (HbA1c/Hb) × 100. The test was standardised with an intraassay coefficient of variation (CV) 0.9%–1.5% and inter-assay CV 1.1%–1.6%. Daily calibration and maintenance of the analyser was performed according to the manufacturer’s instructions using the manufacturer-supplied calibrator (Cfas HbA1c) (18). Quality control was maintained using the quality control materials provided with the analyser by the manufacturer (negative and positive controls [high and low HbA1c]).

Other biochemical parameters such as low-density lipoprotein (LDL), high-density lipoprotein (HDL), triglycerides (TG), total cholesterol (TC), serum bicarbonate, serum creatinine (Cr) and blood urea nitrogen (BUN) were obtained from participants’ previous 2- or 3-months medical records in the hospital.

## Risk of Malnutrition Assessment

Non-biomedical methods were used to access malnutrition risk among participants in this study. Participants’ malnutrition risks were assessed using the Malnutrition Universal Screening Tool (MUST) which relied mainly on anthropometric measurements and presence of acute disease effect or episode which caused or likely to cause nil per os for 5 or more days. MUST is a five-step, easy to use screening tool which health care workers used to identify adults who are malnourished, at risk of malnutrition (under nutrition), or obese while in hospitals, community or in other health care facilities (19). MUST adopt three independent criteria to determine patients’ overall malnutrition risk, which are: i) BMI; ii) unintentional weight loss for the past two or three months and iii) acute disease effect or episode which caused or likely to cause nil per os for ≥ 5 days. Each criterion is rated 0, 1 and 2. Patients’ malnutrition risks are established by summing up all the three criteria to form one global score. A score of 0 represents low risk of malnutrition, medium risk = 1 and high ≥ 2 (19). Each of these criteria can independently predict clinical outcome depending on the clinical circumstance, however, when put together they serve as better predictor than singles.

## Statistical Analysis

The IBM SPSS version 22.0 (SPSS, Chicago, IL, USA) was used to run all statistical analysis in this study. Data normal distribution was checked using Kolmogorov-Smirnov test. Descriptive statistics were used to describe participants’ demographic characteristics, while the one-way ANOVA and post-hoc Bonferroni correction tests used to compare significant mean differences across the three groups of adherence to diabetic feeding recommendation (low, moderate and high). The assumption for using the one-way ANOVA test is that our data came from independent random sample that is normally distributed with homogeneous variance. Multinomial logistic regression models were also used to assess the significant association between adherence to diabetic feeding recommendation with glycaemic control and with malnutrition risk. Also, the assumption for using the multinomial logistic regression models is that the groups (low moderate and high adherence) in our dependent variable ‘diabetics feeding recommendation’ are independent. This assumption states that the choice of or membership in one category is not related to the choice or membership of another category (i.e. the dependent variable). This assumption of independence was tested with the Hausman-McFadden test. Prior to the model building, univariate and bivariate analysis were done to check multicolinearity of our independent variables. Also, multivariate diagnostics (i.e. standard multiple regression) was ran to assess for multivariate outliers for exclusion. The multinomial logistic regression models were built while controlling for significant confounding variables. Continue and categorical variables that are normally distributed were entered into the model as independent variables to determine their predictive associations with the depended variable. The results generated from these models were presented in adjusted odds ratio (AOR) and 95% CI, with variables significant set at 0.05 alpha level.

## Results

General characteristics of participants arepresented in Table 1. Mean (standard deviation [SD]) of total adherence to diabetics feeding recommendation was 32.56 (9.61). Mean (SD) for age, weight and BMI were 58.10 (9.70); 61.70 (9.30) and 23.14 (2.92), respectively. Majority of participants (70.9%) were females, married (64.2%) and live in small towns (76.2%). More than 38% of participants have no formal education, 1.9% have education up to polytechnic, 2.5% have it up to university level and the rest have other form of education. Majority of participants (68.5%) have low malnutrition-risk, 2.5% have moderate risk and 29.1% have high risk.

Participants’ general characteristics across group of adherence to feeding recommendation are shown in Table 2. Weight (*P* = 0.011), total-cholesterol (*P* = 0.003) and HbA1c% (*P*-value < 0.001) were significant with adherence to diabetics feeding-recommendation.

The association of adherence to diabetics feeding recommendation with glycaemic-control (HbA1c% levels), and with malnutrition-risk are shown in Tables 3 and 4. After adjusting for confounders (age, medications intake, physical activity, education, diabetes duration and smoking), low adherence to diabetics feeding recommendation (low following of healthful eating habit) (AOR: 2.56; 95% CI: 1.44, 4.56) was significant with poor glycaemic control (high HbA1c%). Low and moderate adherence to fruit and vegetables (AOR: 2.25; 95% CI: 1.29, 3.91 and AOR: 2.71; 95% CI: 1.48, 4.99, respectively) were significant with poor glycaemic-control (high HbA1c%). Low adherence to whole grain, beans, starchy fruit and plantains (AOR: 3.29; 95% CI: 1.81, 6.02) and low adherence to spacing carbohydrate intake (AOR: 2.63; 95% CI: 1.45, 4.76) were significant with poor glycaemic-control (high HbA1c%). Furthermore, low adherence to cholesterol free oils (walnut, canola, sunflower, safflower, cotton seed, rapeseed or soya bean oil) (AOR: 2.62; 95% CI: 1.49, 4.58) was significant with poor glycemic-control (high HbA1c%).

For malnutrition-risk, high adherence to eating high fat dairy foods (AOR: 0.49; 95% CI: 0.30, 0.82) was significant with high malnutrition-risk. On the other hand, low adherence to eating diets prepared with cholesterol free oils (walnut, canola, sunflower, safflower, cotton seed, rapeseed or soya bean oil) (AOR: 0.54; 95% CI: 0.31, 0.95) and low adherence to lean meat and fish (AOR: 0.54; 95% CI: 0.31, 0.95) were also significant with moderate malnutrition-risk.

## Discussion

We evaluated the association of adherence to diabetics feeding recommendation with glycaemic-control and with malnutrition risk among normal weight persons living with T2DM in Brong Ahafo region, Ghana. Many studies evaluated dietary intakes and diabetes management, but often focus on dietary intake and glycaemic control (HbA1c level). However, since many therapies, including feeding recommendation require that patients discipline themselves in order to change certain behaviours to successfully comply with the recommendation. Patients may not be able to accept this behaviour change and may put up inappropriate behaviours to cope. This, however, may put them at various risk of adverse outcome including malnutrition risk.

In our study we found that generally, participants reported moderate mean of adherence to diabetics feeding recommendation [32.56 (9.61)], and relatively higher means HbA1c% level [8.13 (3.2)] (20). We found that about 29.1% of participants’ have high malnutrition-risk and the rest have moderate and low risk. We also realised that intake of fruits and vegetables below the required servings were significant with glycaemic-control. Participants who reported both low and moderate intake of fruits and vegetable were seemed to have elevated HbA1c%. This finding could possibly be true because low fruits and vegetables intake could correspondingly come with increase intake of carbohydrate for energy in general. When these increased intakes particularly come from refined carbohydrate sources, it could affect blood glucose and make it soars thus increasing HbA1c% level (21). In Sargeant et al. (22) study, participants who reported seldom or no fruits and vegetable intakes were found to have increased HbA1c% level.

The association between diet and type 2 diabetes is well researched by nutrition scientists. Diets characterised by refined grains and high fat are shown to be associated with HbA1c% level in many epidemiological studies (23, 24). Traditionally in Ghana, the main staple food and daily energy source for most Ghanaian, typical comes from whole grain, roots and tubers, and plantain. However, due to nutrition transition, trade policies and economic circumstances, many people have deviated from adhering to these traditional eating pattern but tent to consume more of highly refined carbohydrate foods (25, 26). This phenomenon has affected the general population in Ghana including persons with diabetes. Although dieticians are making cautious efforts to correct this defective eating habit, efforts are not yielding the intended results due to the aforementioned problems. Cost, particularly, has been a major factor militating against adherence to feeding recommendation among persons with diabetes in Ghana. In a focal group discussing Doherty et al. (27), noted that people with diabetes who reported erroneous adherence to feeding recommendation, also reported high cost of fruits and vegetables as major factor militating against their consumption.

Low spacing up of carbohydrates intakes in meals for persons with diabetes were also significant with glycemic control in our study. We found that persons with diabetes who adhere to low carbohydrates spacing in meals were seemed to have poor glycemic control (high HbA1c%). This finding could possibly be true because people with diabetes have impaired glucose metabolism, and therefore, spacing up carbohydrate intakes in meals could help the body appropriately utilise the available glucose in the blood before receiving more. In Hakeem et al. (28) study, people with diabetes while fasting during Ramadan, were found to have nomoglycaemia, when carbohydrate intakes were adequate and spaced up in various meals across the day, these results are consistent with our finding.

Again, we noticed that low and moderate adherence to foods prepared with cholesterol free oils (walnut, canola, sunflower, safflower, fish, cotton seed, rapeseed or soya bean oil) were significant with glycaemic-control. We noticed that low and moderate adherence to these oils seemed to increase poor glycaemic-control (high HbA1c%). This is because insulin plays critical role in lipid metabolism. In cells lipid metabolism, insulin stimulates the hormone lipoprotein lipase to increase the uptake of fatty acid from chylomicrons and very low density lipoprotein. Insulin also stimulates glycolysis which triggered increased glycerol phosphate synthesis and thus enables the body to utilise and remove glucose from the blood. However, during diabetes, the insufficient or impaired insulin functions or both, caused lipids to catabolise and contributes it carbon atoms to the body and thus contribute to rise in blood sugar. This, therefore, explained why low and moderate adherence to foods prepared with cholesterol free oils still seem to increased poor glycaemic-control in our study.

Fish oil was also shown to increase poor control in our study. There are conflicting findings about fish and fish oil consumptions with glycaemic-control among persons with diabetes. In a study evaluating the association between fish oil supplementation and metabolic effects, and impaired glucose intolerance, fish oil supplementation was found to be significant with improved blood glucose and cholesterol levels (29). However, in two separate meta-analysis, fish oil supplementations were not significant with blood glucose level but significantly improve total cholesterol, triglyceride and low density lipoprotein cholesterol (30, 31). Therefore, based on the cardiovascular benefits of fish and fish oil consumption, we collectively add our voices in recommending that patients with diabetes who do not have other medical condition that prevent them from consuming fish and fish oil, should intake these products for their cardiovascular benefits.

We also evaluated the association of adherence to diabetics feeding recommendations with malnutrition risk. We used MUST to assess participants’ malnutrition risk. MUST is a five-steps screening tool for identifying adults who are malnourished, at risk of malnutrition (under nutrition) or obese. It relies on current weight loss, BMI and the present of acute disease episode that caused or likely to cause nil per os for ≥ 5 days to diagnosed malnutrition risk in population. Using this tool and its principles, we found that 29.1% of participants in our study have high malnutrition risk, about 3% have moderate risk and the rest have low malnutrition risk. These finding could be true because physical conditions such as poor dentition, ill-fitting dentures and dysphagia; social conditions such as low income, limited knowledge of diet and cooking skills, alcohol or drug intakes and medical conditions such as eating disorders, altered bowel conditions, dementia and depression could cause patients to have low adherence to healthful eating habits (32). These results could have rippled effect on diabetes management and care because people with diabetes already have increased risk of poor healing and poor health outcomes because of other conditions. Although diabetics feeding recommendation in fact is the healthiest eating style for almost everyone, when people with diabetes, failed to adequately adhere to diabetics feeding recommendation due to the aforementioned problems, they could run at increased risk of malnutrition.

Adherence to eating high fat dairy foods was significant with high malnutrition risk while adherence to cholesterol free oils (walnut, canola, sunflower, safflower, fish, cotton seed, rapeseed or soya bean oil) were significant with moderate malnutrition risk. High fat diets are not recommended to the general population, but to the frail and elderly sick adults, the greatest nutrition risk they faced is malnutrition (under nutrition) rather than overweight or obesity. This is because as people age, generally, their appetite decreased, and this coupled with other physical problems such poor dentition or poor fitting dentures often caused decreased in total foods intake. Fat and oil when added to food generally give flavour and increased peoples’ appetite. In this regard fat and oil in foods could boost the elderly population appetite for food and help them eat enough to decrease the risk of protein-energy malnutrition (inadequate calories intakes) as indicated in our finding. Past research suggested that higher fat and lipids diets could help older adults prevent this forms of malnutrition (33) and this is consistent with our findings.

We also found that low adherence to fish and meat intake was significant with moderate malnutrition risk. Meat and fish provide vital micronutrients such as amino acids, collagen and elastin for muscles and bones development. Meat and fish also provide essential vitamins for bodily function. However, in the absence of energy yielding nutrients such as carbohydrate, meat and fish are converted to energy yielding nutrients for the body. If this negative nutrient balance occurred over a prolonged period, weight lost could occur as a result of the body inability to get essential amino acid collagen and elastin for muscles and bone synthesis. This could, thus, increase the malnutrition risk (proteinenergy malnutrition). In a community based study about foods consumption and malnutrition risk, Jiménez-Redondo et al. (34), noted that intake of meat, fish, dairy products, and fruits and vegetable below desirable amounts were significant with malnutrition risk which is consistent with our results.

## Conclusion and Study Limitations

Our study findings demonstrate that poor adherence to diabetics feeding recommendation could be associated with poor glycaemic controls and malnutrition risk. However, there are some limitations that could not permit us to draw definite conclusion to these effects. Some of these limitations are; we evaluated malnutrition based on anthropometric method using MUST, and therefore cannot conclude that significant association exist between diabetic feeding recommendation and risk of malnutrition thus (micronutrient deficiencies). We, also, employed cross-sectional study and a relatively lower sample size (*n* = 530) to arrive at our findings. Since cross-sectional study method has so many limitations, we cannot say that significant association exist between diabetics feeding recommendation with glycaemic-controls and malnutrition risk. Therefore, moving forward, we recommend that subsequent studies should consider using molecular characterisation to diagnosed malnutrition and also employ more powerful study designs to evaluate these significant associations between diabetics feeding recommendation with glycaemic-controls and malnutrition risk. Notwithstanding these limitations, our study has added knowledge to the literature consenting diabetics feeding recommendation with glycaemic controls and malnutrition risk.

## Figures and Tables

**Table 1:** General characteristics of participant (*n* = 530)

Variable	Mean (SD)	Number (%)
Age (years)	58.10 (9.70)	
Wight (kg)	61.70 (9.30)	
Height (m)	1.63 (0.09)	
BMI (kg/m^2^)	23.14 (2.92)	
Total adherence to diabetics feeding recommendation	32.56 (9.61)	
HbA1c	8.13 (3.20)	
FBS	10.05 (4.55)	
Total cholesterol	7.19 (3.49)	
HDL-cholesterol	1.74 (0.90)	
LDL-cholesterol	5.15 (3.42)	
Triglycerides	4.64 (14.14)	
Systolic BP (mmHg)	135.67 (7.79)	
Diastolic BP (mmHg)	77.79 (12.79)	
Diabetes duration (years)	4.90 (5.40)	
Duration lived with diabetes (years)	4.90 (5.40)	
Sex		
Male		154 (29.1)
Female		376 (70.9)
Marital status		
Married		340 (64.2)
Single		20 (3.8)
Widow		107 (20.2)
Divorce		63 (11.9)
Place of residence		
Village		39 (7.4)
Town		404 (76.2)
City		87 (16.4)
Educational level		
No education		202 (38.1)
Primary		85 (16.0)
Junior High		132 (24.9)
Senior High		67 (12.6)
Training college		21 (4.0)
Polytechnic		10 (1.9)
University		13 (2.5)
Risk of malnutrition		
Low risk		363 (68.5)
Moderate risk		13 (2.5)
High risk		154 (29.1)

**Table 2:** Comparison of participants mean demographic characteristics between the three groups of adherence to diabetics feeding recommendation

Variable	Group of adherence						*F*-statistics (df1, df2)^a^	*P*-value^b^

	Low		Moderate		High			
	*n*	Mean (SD)	*n*	Mean (SD)	*n*	Mean (SD)		
Height (m)	181	1.63 (0.08)	175	1.63 (0.08)	174	1.63 (0.09)	0.453 (2,527)	0.636
Weight (kg)	181	60.01 (9.47)	175	**62.52 (9.29)**	**174**	**62.59 (8.80)**	**4.560** (2,527)	**0.011**
BMI (kg/m^2^)	181	22.58 (2.81)	175	23.47 (2.96)	**174**	**23.39 (2.92)**	**5.235** (2,527)	**0.006**
Systolic BP (mmHg)	181	134.31 (21.68)	175	137.41 (19.59)	174	135.32 (19.96)	1.057 (2,527)	0.348
Diastolic BP (mmHg)	181	77.32 (12.36)	175	78.87 (12.44)	174	77.19 (13.57)	0.935 (2,527)	0.393
Diabetes duration (years)	181	4.48 (2.98)	175	4.75 (2.99)	174	5.03 (3.07)	1.493 (2,527)	0.226
Age (years)	181	58.63 (10.06)	175	57.11 (10.01)	174	58.40 (8.78)	1.268 (2,527)	0.282
**Total cholestrol (mg/dL)**	181	6.88 (3.39)	175	**6.36 (3.39)**	**174**	**7.97 (3.64)**	**5.754** (2,527)	**0.003**
HDL cholesterol (mg/dL)	181	1.81 (0.98)	175	1.71 (0.89)	174	1.69 (0.82)	0.985 (2,527)	0.374
LDL cholestrol (mg/dL)	181	5.43 (3.45)	175	5.08 (3.84)	174	4.94 (2.86)	0.993 (2,527)	0.371
Triglyceride (mg/dL)	181	4.11 (11.71)	175	5.42 (16.75)	174	4.39 (13.63)	0.417 (2,527)	0.659
HbA1c (%)	181	9.58 (3.79)	175	**7.69 (2.51)**	**174**	**7.07 (2.61)**	**32.890** (2,527)	**0.0001**
Fasting blood sugar (mmol/L)	181	11.59 (5.28)	175	9.69 (4.14)	**174**	**8.81 (3.60)**	**18.563** (2,527)	**0.0001**

Notes:

a One-way ANOVA;

b Post-hoc analysis with Bonferroni correction that show significant mean difference in: i) weight between low adherence and other adherence to recommended dietary guideline (*P* = 0.011 ), ii) BMI between low adherence and other adherence to recommended dietary guideline (*P* = 0.006), iii) Total-cholesterol between low adherence and other adherence to recommended dietary guideline (*P* = 0.003), iv) Fasting blood sugar between low adherence and other adherence to recommended dietary guideline (*P* = 0.0001)

**Table 3:** Association of adherence to diabetic feeding recommendation with glycaemic control

Variable		Moderate (HbA1c%)		High (HbA1c%)		Moderate (HbA1c%)		High (HbA1c%)	

		Crude OR (95% Cl)	*P-value	Crude OR (95% CI)	*P-value	AOR (95% Cl)	**P-value	AOR (95% Cl)	**P-value
**Follow healthful eating habit 3**	**Ref**	**1**		**1**		**1**		**1**	
Follow healthful eating habit 2		1.41 (0.82, 2.41)	0.211	1.72 (0.96, 3.10)	0.069	1.41 (0.82, 2.43)	0.214	1.69 (0.94, 3.08)	0.081
Follow healthful eating habit 1		1.02 (0.58, 1.79)	0.950	**2.61 (1.48, 4.58)**	**0.001**	0.99 (0.56, 1.76)	0.974	**2.56 (1.44, 4.56)**	**0.001**
**Eat require fruit and vegetable 3**	**Ref**	**1**		**1**		**1**		**1**	
Eat required fruit and vegetable 2		1.28 (0.77, 2.12)	0.350	**2.18 (1.27, 3.74)**	**0.004**	1.32 (0.78, 2.21)	0.300	**2.25 (1.29, 3.91)**	**0.004**
Eat required fruit and vegetable 1		0.89 (0.48, 1.66)	0.716	**2.57 (1.42, 4.66)**	**0.002**	0.90 (0.48, 1.69)	0.745	**2.71 (1.48, 4.99)**	**0.001**
**Eat whole grain, beans, starchy fruits and plantain 3**	**Ref**	**1**		**1**		**1**		**1**	
Eat whole grain, beans, starchy fruits and plantain 2		1.04 (0.62, 1.73)	0.896	1.39 (0.81, 2.41)	0.229	1.08 (0.65, 1.82)	0.760	1.47 (0.84, 2.55)	0.177
Eat whole grain, beans, starchy fruits and plantain 1		1.21 (0.65, 2.24)	0.545	**3.28 (1.81, 5.91)**	**0.001**	1.19 (0.64, 2.23)	0.571	**3.29 (1.81, 6.02)**	**0.001**
**Space carbohydrate intake 3**	**Ref**	**1**		**1**		**1**		**1**	
Space carbohydrate intake 2		1.20 (0.69, 2.09)	0.521	1.53 (0.84, 2.81)	0.166	1.27 (0.72, 2.25)	0.404	1.73 (0.93, 3.21)	0.082
Space carbohydrate intake Adj1		1.25 (0.71, 2.19)	0.435	**2.54 (1.42, 4.53)**	**0.002**	1.33 (0.75, 2.34)	0.332	**2.63 (1.45, 4.76)**	**0.001**
**High fat dairy foods 1**	**Ref**	**1**		**1**		**1**		**1**	
Eat high fat dairy foods 2		0.98 (0.50, 1.92)	0.942	0.81 (0.40, 1.63)	0.547	0.91 (0.46, 1.82)	0.794	1.09 (0.66, 1.79)	0.741
Eat high fat dairy foods 3		1.13 (0.69, 1.85)	0.639	1.13 (0.69, 1.85)	0.639	1.11 (0.67, 1.82)	0.691	0.729 (.36, 1.49)	0.390
**Eat food prepared with walnut, canola sunflower, safflower, cotton seed, fish or soya oil**	**Ref**	**1**		**1**		**1**		**1**	
Eat food prepared with walnut, canola sunflower, safflower, cotton seed, fish or soya oil		**2.10 (1.21, 3.65)**	**0.008**	**2.56 (1.40, 4.66)**	**0.002**	**2.23 (1.27, 3.90)**	**0.005**	**2.75 (1.49, 5.09)**	**0.001**
Eat food prepared with walnut, canola sunflower, safflower, cotton seed, fish oil 1		0.84(0.48, 1.47)	0.538	**2.60 (1.51, 4.47)**	**0.001**	0.83 (0.47, 1.48)	0.531	**2.62 (1.49, 4.58)**	**0.001**
**Intake lean meat and fish 3**	**Ref**	**1**		**1**		**1**		**1**	
Intake lean meat and fish 2		0.84 (0.42, 1.66)	0.614	1.15 (0.56, 2.35)	0.699	0.79 (0.45, 1.42)	0.441	1.08 (0.59, 1.96)	0.798
Intake lean meat and fish Adj1		1.08 (0.57, 2.04)	0.812	**2.35 (1.21, 4.57)**	**0.012**	0.89 (0.52, 1.55)	0.694	1.73 (0.99, 2.97)	0.052

Notes: Ref = as reference;

* *P*-value results from univariate analysis;

** *P*-value results from multinomial logistic regression model after adjusting for age, diabetes duration, education, diabetes medications intake, smoking and physical activity; *n* = 530

**Table 4:** Association adherence to diabetic feeding recommendation with risk of malnutrition

Variable		Moderate (malnutrition risk)		High (malnutrition risk)		Moderate (malnutrition risk)		High (malnutrition risk)	

		Crude OR (95% Cl)	*P-value	Crude OR (95% CI)	*P-value	AOR (95% Cl)	** *P*-value	AOR (95% Cl)	**P-value
**Follow healthful eating habit 3**	**Ref**	**1**		**1**		**1**		**1**	
Follow healthful eating habit 2		1.25 (0.73, 2.13)	0.416	1.03 (0.61, 1.72)	0.919	1.37 (0.75, 2.50)	0.312	1.43 (0.81, 2.57)	0.225
Follow healthful eating habit 1		0.81 (0.48, 1.34)	0.412	**0.48 (0.29, 0.79)**	**0.005**	0.95 (0.53, 1.69)	0.863	0.74 (0.41, 1.29)	0.278
**Eat required fruit and vegetable Adj 3**	**Ref**	**1**		**1**		**1**		**1**	
Eat require fruit and vegetable 2		1.08 (0.66, 1.75)	0.773	0.91 (0.56, 1.48)	0.706	1.25 (0.73, 2.14)	0.412	0.92 (0.55, 1.55)	0.748
Eat required fruit and vegetable 1		0.64 (0.38, 1.08)	0.096	**0.52 (0.30, 0.88)**	**0.015**	0.97 (0.54, 1.77)	0.930	0.63 (0.35, 1.15)	0.130
**Eat whole grain, beans, roots and plantain 3**	**Ref**	**1**		**1**		**1**		**1**	
Eat whole grain, beans, roots and plantain 2		0.74 (0.45, 1.22)	0.242	0.79 (0.49, 1.29)	0.348	0.81 (0.47, 1.39)	0.447	0.95 (0.56, 1.60)	0.839
Eat whole grain, beans, roots, and plantain 1		1.00 (0.59, 1.67)	0.999	0.72 (0.43, 1.23)	0.232	1.05 (0.59, 1.88)	0.857	0.86 (0.48, 1.54)	0.621
**Space carbohydrate intake Adj3**	**Ref**	**1**		**1**		**1**		**1**	
Space carbohydrate intake 2		1.28 (0.75, 2.19)	0.367	0.75 (0.44, 1.27)	0.281	1.42 (0.77, 2.63)	0.261	0.81 (0.45, 1.45)	0.479
Space carbohydrate intake 1		1.20 (0.71, 2.05)	0.499	0.76 (0.45, 1.26)	0.283	1.23 (0.68, 2.23)	0.487	0.82 (0.47, 1.43)	0.479
**Eat high fat dairy foods Adj1**	**Ref**	**1**		**1**		**1**		**1**	
Eat high fat dairy foods 2		1.14 (0.59, 2.19)	0.707	0.99 (0.52, 1.89)	0.993	1.11 (0.53, 2.33)	0.790	1.03 (0.50,2.12)	0.937
Eat high fat dairy foods 3		0.80 (0.50, 1.28)	0.348	**0.55 (0.34, 0.87)**	**0.010**	0.65 (0.39, 1.08)	0.094	**0.49 (0.30, 0.82)**	**0.006**
**Eat food prepared with walnut, canola sunflower, safflower, cotton seed, fish or soya oil 3**	**Ref**	**1**		**1**		**1**		**1**	
Eat food prepared with walnut, canola sunflower, safflower, cotton seed, fish or soya oil 2		0.89 (0.53, 1.50)	0.669	0.77 (0.45, 1.29)	0.322	0.76 (0.43, 1.35)	0.353	0.86 (0.49, 1.51)	0.589
Eat food prepared with walnut, canola sunflower, safflower, cotton seed, fish or soya oil 1		0.61 (0.37, 1.02)	0.057	**0.59 (0.36, 0.97)**	**0.039**	**0.54 (0.31, 0.95)**	**0.034**	0.69 (0.40, 1.19)	0.187
**Intake lean meat and fish 3**	**Ref**	**1**		**1**		**1**		**1**	
Intake lean meat and fish 2		1.25 (0.77, 2.04)	0.361	0.92 (0.57, 1.49)	0.736	1.38 (0.80, 2.37)	0.247	1.05 (0.62, 1.78)	0.851
Intake lean meat and fish 1		**1.85 (1.08, 3.16)**	**0.025**	1.36 (0.79, 2.32)	0.264	**2.09 (1.14, 3.86)**	**0.017**	1.60 (0.89, 2.89)	0.120

Notes: Ref = as reference;

* *P*-value results from univariate analysis;

** *P*-value results from multinomial logistic regression model after adjusting for age, diabetes duration, education, diabetes medications intake, smoking and physical activity; *n* = 530
